# Pancreatic cancer presenting as bowel obstruction and role of next generation sequencing: A case report

**DOI:** 10.1016/j.ijscr.2021.106654

**Published:** 2021-12-03

**Authors:** Bach Ardalan, Jose Azqueta, Jonathan England, Rene Hartmann

**Affiliations:** a Sylvester Comprehensive Cancer Center, United States of America; b Baptist Hospital of Miami, United States of America

**Keywords:** Pancreatic adenocarcinoma, Colonic metastasis, Colonic obstruction, Next-generation-sequencing, Case report

## Abstract

**Introduction and importance:**

Pancreatic adenocarcinoma is one of the leading causes of death. Presentation with colonic metastases is far less frequently reported in the literature and may be misdiagnosed as colonic adenocarcinoma. We report the case of a female patient with metastatic pancreatic adenocarcinoma that presented with a sigmoid obstruction.

**Case presentation:**

A 66-year-old female presented with constipation and abdominal pain. She was found to have an obstructing sigmoid colon lesion, multiple metastatic lesions in the liver, and a pancreatic tail lesion. She underwent left hemicolectomy and ostomy placement. The gross pathology of the colon and needle biopsy of the liver was consistent of pancreatobiliary origin. Genomic screening performed, patient found to be KRAS G12R mutated. She was given one cycle of chemotherapy, thereafter was referred to hospice care.

**Clinical discussion:**

Primary metastatic pancreatic cancer is now the 2nd most diagnosed cancer in the United States after lung cancer. The prognosis for the malignancy is poor, patients are usually diagnosed late at the time that the tumor has metastasized to other organs. Colonic metastasis is a rarely seen and far less frequently reported in the literature. Next-generation-sequencing was performed at baseline to further characterize her tumor for any actionable mutations.

**Conclusion:**

Pancreatic adenocarcinoma is an aggressive malignancy with a poor prognosis. Next-generation-sequencing may offer targeted therapy if an actionable mutation is present such as our patient's, however due to late diagnosis, rapid clinical deterioration, and next-generation sequencing delay we were unable to alter the patient's outcome.

## Highlights

• Colonic obstruction is a rare presentation of metastatic pancreatic adenocarcinoma.
• Next-generation sequencing may offer therapies if patient has a targetable mutation.
• Pancreas cancer is limited in treatment, NGS may offer better outcome if targetable.

## 1 Case presentation

A 66-year-old female, with no prior pertinent personal or family medical history, presented to a local hospital in Venezuela following episodes of constipation and abdominal pain. She had no pertinent medical history prior to this presentation. The patient was found to have an obstructing lesion at the sigmoid colon, multiple hepatic lesions, and a pancreatic tail lesion. She underwent emergency laparoscopic left hemicolectomy with creation of an end-colostomy. Surgical pathology indicated the sigmoid lesion to be moderately-differentiated adenocarcinoma (4 × 2 × 2 cm in size). Margins were negative. The hepatic metastases were biopsied and were found to be consistent with adenocarcinoma of pancreatic origin. In Venezuela, she received two cycles of attenuated FOLFIRINOX, which were reportedly well tolerated.

**Fig. 1:**
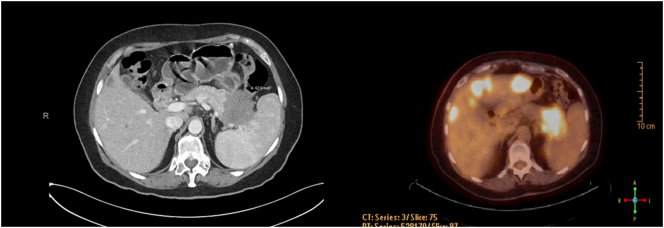
Pancreatic Tail lesion (CT with contrast and PET/CT, side by side). The prominent pancreatic tail mass invades into the splenic hilum, stomach, and splenic flexure, encasing the splenic vessels.

**Fig. 2:**
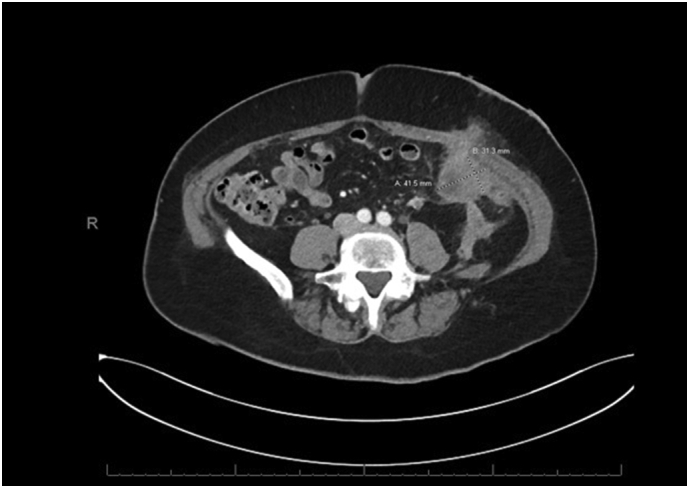
Prominent omental lesion adjacent to the site of stoma.

**Fig. 3:**
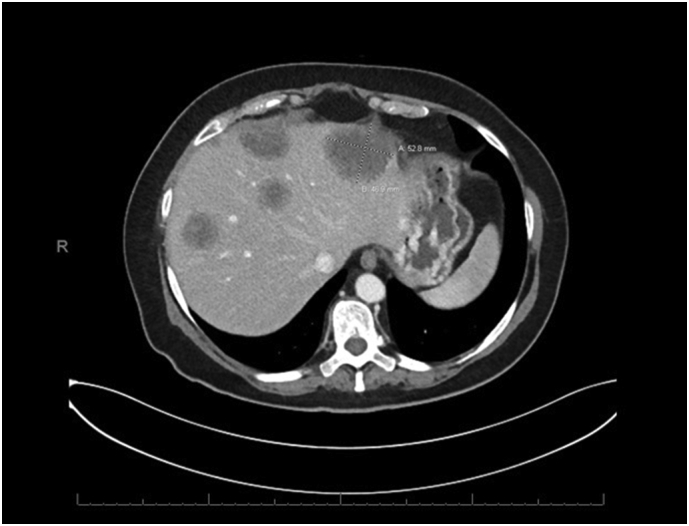
Multiple hepatic metastases, previously biopsied confirmed to of pancreatobiliary primary.

Soon thereafter she transferred her care to the United State**s** and was seen in outpatient clinic. The pathology slides were reviewed, and the diagnosis was confirmed to be moderately to poorly differentiated adenocarcinoma consistent with a pancreatobiliary primary. Original imaging studies were not available. A CT Chest Abdomen with contrast indicated a prominent pancreatic tail mass invading the splenic hilum, stomach, and splenic flexure, encasing the splenic vessels (Fig. 1). Multiple omental lesions were seen, notably one adjacent to her stoma site (Fig. 2). Multiple hepatic lesions visualized (Fig. 3). A liquid biopsy (Guardant360) was sent out, and she was found to have the following targetable mutations: FGFR amplification, BRCA D1923N, and a KRAS G12R variant. Her case was discussed in tumor board. She was given one cycle of FOLFIRINOX at full dose, however, this was poorly tolerated. Clinical status continued to decline, and subsequently the patient elected not to have any further chemotherapy. Thereafter, the family's decision was to enroll the patient into hospice care.

## 2 Pathology and immunohistochemistry

**Fig. 4:**
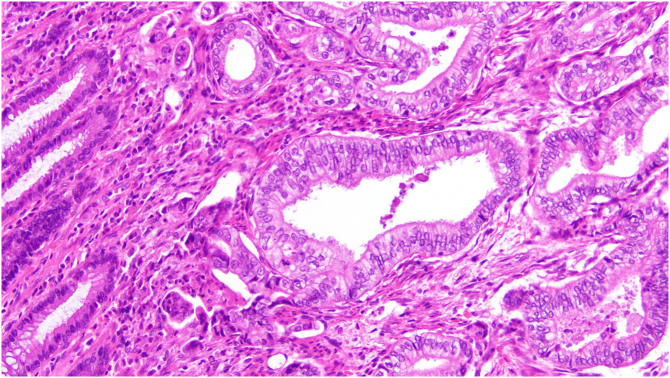
Pathological Analysis of Colonic Metastases: Adenocarcinoma found in sigmoid colon (×20 magnification).

**Fig. 5:**
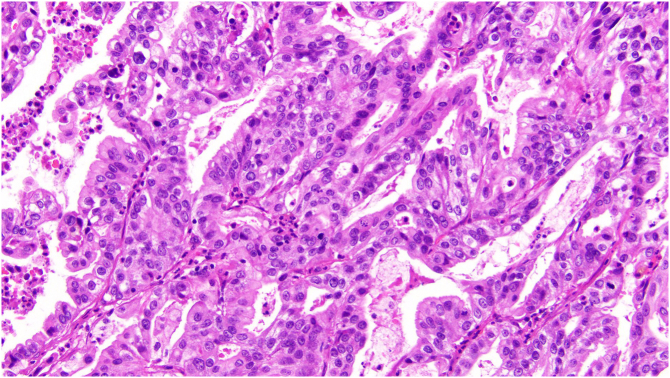
Pathological Analysis of Colonic Metastases: Adenocarcinoma found within hepatic parenchyma (×20 magnification).

Sigmoidal lesion (Fig. 4) and liver metastases (Fig. 5) were confirmed to be adenocarcinoma of pancreatobiliary origin despite the presence of neoplasm in regional lymph nodes in the colonic specimen. Immonustaining results indicated positive for CK7 staining and negative for CK20- and SATB2 staining. Immunohistochemistry for DNA mismatch repair (MMR) proteins performed indicated MLH1, MSH2, MSH6, PMS2.

## 3 Discussion

Pancreatic cancer is often diagnosed at late stages and often metastasized to other organs thus surgery is not an option. Our patient presented with a sigmoid obstruction, initially it was thought to be colon cancer, however, following pathological review it was confirmed that it was a metastasis from her pancreatic adenocarcinoma.

As the rate of pancreatic cancer has steadily increased over the past decade, so has its presentations with colonic metastases. Synchronous presentation of pancreatic cancer with colonic metastases are rare, with only a few reported cases thus far in the literature.

In the literature, there are few reported cases of pancreatic cancer metastasizing to the colon. The first reported case in the literature of synchronous metastasis to the colon presented as an ascending colon lesion. The colonic mass was resected and determined to be of pancreatic origin. No further follow up was given (Bellows et al., 2009) [1].

The second case reported in the literature of synchronous colonic metastases of a pancreatic primary is that of a 67-year-old female with worsening abdominal pain and constipation. At presentation she was found to have a lesion on the distal sigmoid colon, peritoneal nodules and mass at the tail of the pancreas. She underwent resection of the peritoneal implant and colectomy with the creation of an end colostomy. Although the patient was initiated on FOLFIRINOX chemotherapy, she did not tolerate the first round and thereafter discharged home on hospice [2].

The third reported case in the literature describes a 60-year-old male who presented with thickening of the sigmoid colon, pancreatic tail mass involving the spleen and left kidney, multiple mesenteric masses, suggesting metastatic disease. Biopsy of an umbilicial nodule revealed metastases from pancreatic adenocarcinoma. The patient underwent palliative FOLFOX chemotherapy however his colonic lesion perforated, thereafter patient was admitted for emergency surgery. He continued to receive palliative treatment and was died 9 months after his diagnosis [3].

The fourth reported cased reported a 91-year-old patient who presented with 13.6 kg weight loss and a long-reported history of constipation. She underwent sigmoidoscopy; however stenosis was found in the sigmoid colon and unable to be traversed. She underwent palliative sigmoid colectomy with end colostomy. Thereafter a CT scan was performed revealing a pancreatic head mass. Pathology of the metastatic lesion revealed well-differentiated adenocarcinoma with CK7 positive and CK20 negative staining. CA19-9 was elevated. Patient desired comfort care and discharged home with hospice care [4].

The fifth reported case in the literature was that of a 71-year-old male with synchronous descending colon/sigmoid lesion, elevated CA19-9. Lesion was biopsied and immunhistochemistry showed positive CK7 staining and only few cells of CK20, thereby confirming pancreatic origin. Thereafter the patient initiated palliative abraxane/gemzar chemotherapy. No further follow up indicated [5].

The final reported case in the literature up to the present is that of a 74-year-old male patient who presented to his local ED with tarry stools and significant lethargy. Baseline imaging was noncontributory, and the patient was admitted. Upon admission the patient underwent colonoscopy which revealed a lesion at approximately 70 cm. Mass prevented further examination, and patient underwent left hemicolectomy, splenectomy and partial distal pancreatectomy. Pathology demonstrated pancreatic ductal adenocarcinoma with extension into wall of the splenic flexure. It was noted that the patient's CA19-9 was elevated and the carcinoma invaded the peripancreatic soft tissue [6].

In our patient, a liquid biopsy was performed, in order to determine any targetable mutations. Among the ones reported, her tumor harbored a KRAS G12R mutation. The KRAS oncogene is involved in the MAPK/ERK pathway in functions such as cell differentiation, proliferation, and cell death. KRAS G12R is most commonly found in pancreatic cancer, totaling 15% overall in comparison to the more frequent G12D and G12V mutations [7]. Furthermore, patients who have this mutation tend to have a better overall prognosis in comparison to G12D [8]. In our previous studies, KRAS G12R mutated patients have showed benefit to addition of MEK inhibitors alongside chemotherapy [9]. Unfortunately, baseline next-generation sequencing may take one to three months.

## 4 Conclusion

Metastases from pancreatic adenocarcinoma to the sigmoid colon is a rare, but it has been noted in the literature. Although our patient was not well enough for further chemotherapy, she may have benefited from the possible combination of chemotherapy and a MEK inhibitor given her KRAS G12R mutation. In addition to this, whole genome analysis has allowed for the development of targeted therapies, indicating added benefit of baseline molecular sequencing of the patient's tumor. We believe it is pertinent for oncologists to order next-generation sequencing on the patient's tumor at the time of the initial visit since typically the result of the tests are not available for several months.

This paper was reported in line with the SCARE 2020 Guidelines [10].

## Human subjects

Written informed consent was obtained from the patient.

## Financial relationships

All authors have declared that they have no financial relationships at present or within the previous five years with any organizations that might have an interest in the submitted work. This research did not receive any specific grant from funding agencies in the public, commercial, or not-for profit sectors.

## Other relationships

All authors have declared that there are no other relationships or activities that could appear to have influenced the submitted work.

## Ethical approval

Research studies involving patients require ethical approval. Please state whether approval or exemption has been given, name the relevant ethics committee and the state the reference number for their judgement. Please give a statement regarding ethnical approval that will be included in the publication of your article, if the study is exempt from ethnical approval in your institution please state this.

## Consent

Written informed consent was obtained from the patient. A copy of the written consent is available for review by the Editor-in-Chief of this journal on request.

## Guarantor

Bach Ardalan M.D.

## Provenance and peer review

Not commissioned, externally peer-reviewed.

## CRediT authorship contribution statement

All authors were equally involved in writing the manuscript.

## Declaration of competing interest

In compliance with the ICMJE uniform disclosure form, all authors do not report any conflict of interest.
